# Coordinated reset neuromodulation for Parkinson's disease: Proof-of-concept study

**DOI:** 10.1002/mds.25923

**Published:** 2014-06-28

**Authors:** Ilya Adamchic, Christian Hauptmann, Utako Brigit Barnikol, Norbert Pawelczyk, Oleksandr Popovych, Thomas Theo Barnikol, Alexander Silchenko, Jens Volkmann, Günter Deuschl, Wassilios G Meissner, Mohammad Maarouf, Volker Sturm, Hans-Joachim Freund, Peter Alexander Tass

**Affiliations:** 1 Institute of Neuroscience and Medicine-Neuromodulation, Jülich Research CenterJülich, Germany; 2 Department of Neuromodulation, University of CologneCologne, Germany; 3 Department of Child- and Adolescent Psychiatry, University of CologneCologne, Germany; 4 Clinic for Stereotactic and Functional Neurosurgery, University of CologneCologne, Germany; 5 Department of Neurology, University WürzburgWürzburg, Germany; 6 Department of Neurology, University KielKiel, Germany; 7 Institut des Maladies Neurodégénératives, Univ. de BordeauxBordeaux, France; 8 CNRS Institut des Maladies NeurodégénérativesBordeaux, France; 9 Service de Neurologie, CHU de BordeauxPessac, France; 10 Centre de référence atrophie multisystématisée, CHU de BordeauxPessac, France

**Keywords:** Parkinson's disease, coordinated reset neuromodulation, DBS, beta band oscillation, STN

## Abstract

**Background:**

The discovery of abnormal synchronization of neuronal activity in the basal ganglia in Parkinson's disease (PD) has prompted the development of novel neuromodulation paradigms. Coordinated reset neuromodulation intends to specifically counteract excessive synchronization and to induce cumulative unlearning of pathological synaptic connectivity and neuronal synchrony.

**Methods:**

In this prospective case series, six PD patients were evaluated before and after coordinated reset neuromodulation according to a standardized protocol that included both electrophysiological recordings and clinical assessments.

**Results:**

Coordinated reset neuromodulation of the subthalamic nucleus (STN) applied to six PD patients in an externalized setting during three stimulation days induced a significant and cumulative reduction of beta band activity that correlated with a significant improvement of motor function.

**Conclusions:**

These results highlight the potential effects of coordinated reset neuromodulation of the STN in PD patients and encourage further development of this approach as an alternative to conventional high-frequency deep brain stimulation in PD. © 2014 The Authors. *Movement* Disorders published by Wiley Periodicals, Inc. on behalf of International Parkinson and Movement Disorder Society.

Deep brain stimulation (DBS) of the subthalamic nucleus (STN) is a well-established treatment for patients with advanced Parkinson's disease (PD),^1^,^2^ Novel stimulation approaches, for instance, closed-loop neurostimulation that have only been tested in an acute setting, turned out to be more effective than classical DBS in reducing motor signs as well as pallidal firing rate and oscillatory activity in parkinsonian nonhuman 1-methyl-4-phenyl-1,2,3,6-tetrahydropyridine (MPTP)-treated primates and PD patients during stimulus delivery.^3^,^4^

Another original approach, electrical coordinated reset (CR) neuromodulation,^5^,^6^ specifically targets PD-related pathological neuronal synchrony^7^,^8^ by desynchronization and is based on extensive computational^5^,^6^ and in vitro^9^ studies. Coordinated reset neuromodulation means to consecutively deliver brief high-frequency pulse trains through the different stimulation contacts of the implanted lead (see Supplemental Data Fig. S1) to sequentially reset the phases of the different stimulated subpopulations and, hence, divide the neuronal population into phase-shifted subpopulations, ultimately causing an unlearning of both pathological neuronal synchrony and pathological synaptic connectivity.^5^,^6^,^9^ According to the model, CR neuromodulation of sufficient duration is expected to shift the neuronal population into a stable desynchronized state, characterized by downregulated synaptic connectivity. Accordingly, one may speculate that neuronal desynchronization along with positive effects on motor control may outlast the CR neuromodulation duration.

In both MPTP-treated monkeys^10^ and PD patients,^11^ only short-lasting aftereffects are observed when conventional high-frequency neurostimulation is terminated. In parallel, abnormal oscillatory activity and neuronal synchrony reemerge shortly after turning off classical DBS.^8^,^12^ In contrast, in MPTP-treated nonhuman primates, CR neuromodulation delivered to the STN for 2 hours per day on 5 consecutive days had both acute and sustained long-lasting aftereffects on motor function for up to 30 days.^10^

As yet, physiological aftereffects of CR neuromodulation of the STN on abnormal neuronal synchrony were studied neither in MPTP monkeys nor in PD patients, and a clinical proof-of-concept is lacking in humans. Here we aimed to assess (1) initial safety and tolerability of electrical CR neuromodulation, (2) initial estimation of the action of electrical CR neuromodulation on the abnormal local field potential (LFP) oscillations, (3) initial estimation of the action of electrical CR neuromodulation on motor function of patients with Parkinson's disease in a small proof-of-concept study of short duration. Specifically, we hypothesize that CR neuromodulation causes a lasting reduction of abnormal power in beta or theta frequency bands in parallel with a reduction of motor symptoms.^5^,^6^,^10^ Results of this study will provide information necessary regarding potential trial endpoints and therapeutic regimen(s) for future controlled trials of electrical CR neuromodulation.

## Methods

Six patients (4 women and 2 men) aged 45 to 73 years (mean ± standard deviation, 61.3 ± 11.5 years) with PD (2 with akinetic-rigid and 4 with equivalent type; see Supplemental Data for the definition; mean ± standard deviation disease duration, 11.3 ± 5.8 years) were selected for the study. The mean Unified Parkinson's Disease Rating Scale (UPDRS) motor score (sum of the items 18-31, on medication) was 33.3 ± 5.3 in the presurgery phase.^13^ The local ethical committee approved the study design, and all patients gave written informed consent. We set out to investigate electrophysiological and neurological outcomes of electrical CR neuromodulation of the STN in this cohort of six PD patients.

**Figure 1:**
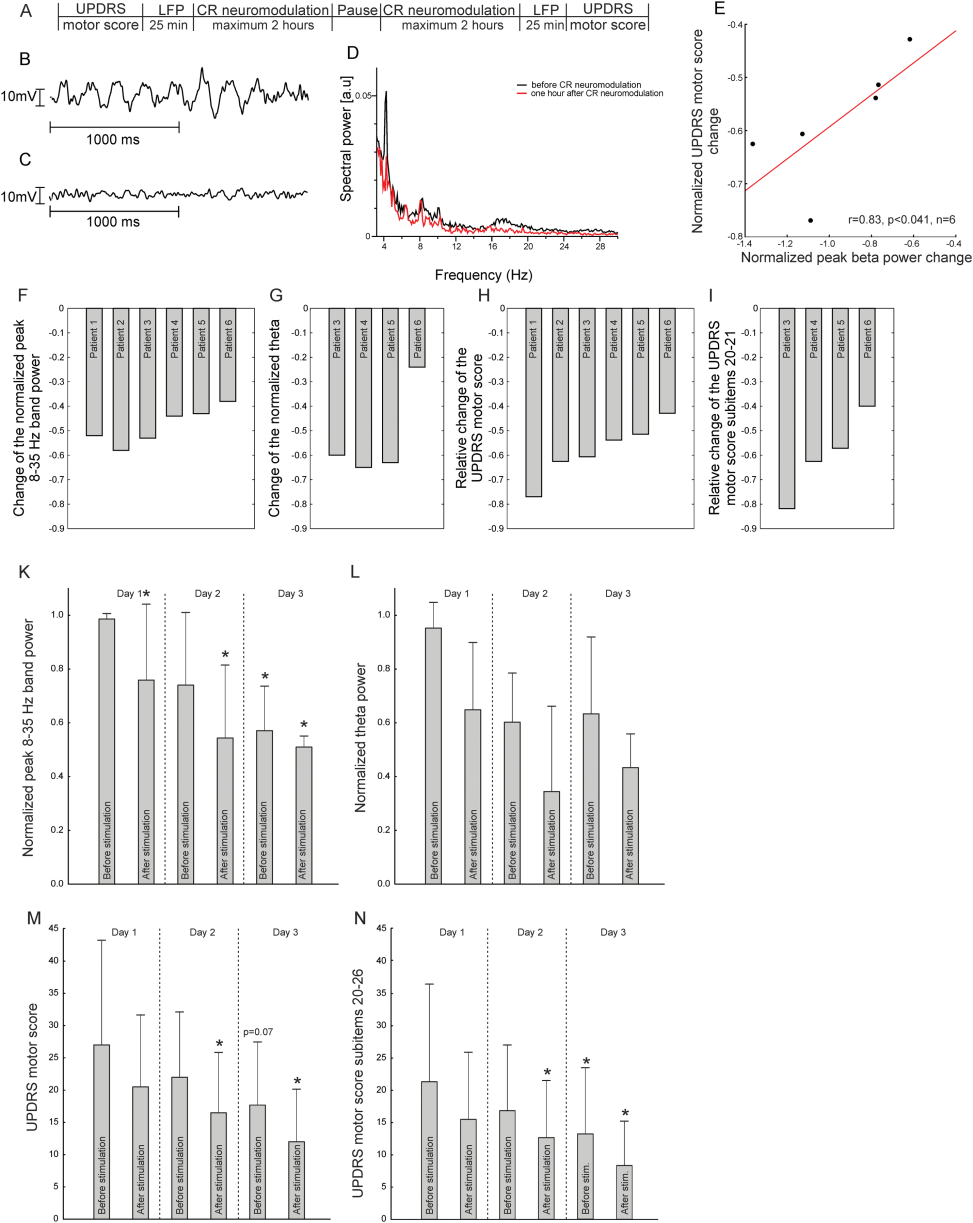
Effect of CR neuromodulation on normalized LFP activity and motor performance in six PD patients. Coordinated reset neuromodulation and measurements followed the experimental scheme in (A) on each of the 3 stimulation days. Example of the raw LFP signal at baseline (B, first day before stimulation) and after 3 days of CR neuromodulation (C, in the evening of the third day of stimulation) and the LFP power spectrum (D) obtained from the patient 3 where an extended spontaneous LFP was obtained before and 1 hour after cessation of CR neuromodulation illustrate the effect of CR neuromodulation. The normalized reduction of UPDRS motor scores and the reduction of the averaged beta band power obtained at the third stimulation day (E, after stimulation) were positively correlated. Effects of CR neuromodulation on normalized individual LFP activity and individual motor performance in six PD patients (F, G, H, I) on the evening of the third day of stimulation. Patients 1 and 2 did not show tremor, and therefore theta band activity was not analyzed in these two patients. The averaged normalized beta band power (K), the average normalized theta band power (L, obtained from the 4 patients with tremor), as well as UPDRS motor score (items 18-31) (M) and UPDRS motor score subitems 20 through 26 (N), improved cumulatively and consistently over 3 days of stimulation. Wilcoxon matched-pairs test was performed to evaluate changes of LFP activity and scores. Significant results are marked by the star (*P* < 0.05). Please note, because of the small sample size of this exploratory proof of concept study, the statistical analysis has limited explanatory power. Therefore, the beneficial clinical effects observed in all subjects consistently are of more importance. Abbreviations: CR, coordinated reset; LFP, local field potential; UPDRS, Unified Parkinson's Disease Rating Scale.

All patients underwent bilateral implantation of quadripolar deep-brain electrodes (Medtronic 3389) into the STN, and stimulation was applied through the externalized electrodes, using a portable external stimulation and registration device.^14^ Medication was suspended 2 days before starting test stimulation and throughout the entire testing period. Coordinated reset neuromodulation^5^ was applied for 3 consecutive days in two daily sessions of up to 2 hours (mean duration of each sessions was 1.6 ± 0.3 hours; Fig. 1A) and consisted of brief high-frequency pulse trains (intra-burst frequency: 130 Hz, intensity: 2.0-4.0 mA, cycle repetition rate 3-20 Hz, pulse width: 60-120 µs, each pulse train was composed of 3 to 5 single pulses, pulse train duration was 23-38 ms) that were applied following the dedicated CR pattern (see Supplemental Data Fig. S1) through the three lower contacts of the stimulation electrode, and the upper contact served as current return. Because of technical constraints, CR neuromodulation was performed unilaterally, exclusively contralateral to the more severely affected side.

Standardized evaluations using UPDRS motor scores were undertaken in all patients 25 minutes before (morning scores) and 25 minutes after (evening scores) stimulation sessions (Fig. 1A) on every stimulation day to assess motor performance before and after CR neuromodulation. The LFPs of the STN were recorded before and after stimulation sessions on every stimulation day according to the scheme presented in Fig. 1A. We calculated the individual peak beta power (in the 8-35 Hz range), which is believed to be related to bradykinesia and rigidity^7^,^8^ and individual theta power (3-7 Hz), which corresponds to tremor frequency.^15^ The spectral power values were then normalized for every patient separately and finally averaged over all six patients.

Acute after-effects on beta (or correspondingly theta) band activity and UPDRS motor scores achieved within a single stimulation day were calculated with the formula: (morning beta power − evening beta power)/morning beta power. To assess lasting aftereffects, that is, effects persisting overnight, we determined relative changes of quantities measured in the morning of days 2 and 3 with respect to the baseline value obtained in the morning of day 1, for example, (baseline beta − beta in the morning of day 2)/baseline beta. In addition, if a repeated administration of CR neuromodulation produces effects that are more pronounced than those produced by the first daily CR neuromodulation dose, such as leading to better improvement in the evening on day 3 as opposed to the evening on day 1, we term these effects cumulative. A Wilcoxon matched pairs test was performed to evaluate changes in LFP activity and UPDRS scores. See Supplemental Data for a detailed description of the methods.

## Results

All six enrolled subjects received CR neuromodulation during 3 stimulation days. The averaged peak beta power decreased gradually over the 3 stimulation days, resulting in a mean reduction of 42.0%, *P* = 0.03/48.0%, *P* = 0.03 in the morning/evening of day 3 relative to baseline in all six patients (Fig. 1F, K; Table1). Acute CR-induced effects were already observed on day 1 in five patients (mean reduction, 22.8%; *P* = 0.046, n = 6; Fig. 1K, Table1), with a mean acute aftereffect on the peak beta band power averaged over the 3 stimulation days of 16.1% (*P* = 0.04). Effects of CR neuromodulation on the LFP theta band power are presented in Figure 1G, L, and Table1. The acute theta change averaged over the 3 stimulation days was 32.1% (*P* = 0.02).

**Table 1:** Absolute and relative changes in beta and theta activity and UPDRS motor scores in 6 patients over the 3-d period of CR neuromodulation^a^

		Day 1		Day 2		Day 3	
		Morning assessment (before stimulation)	Evening assessment (after stimulation)	Morning assessment (before stimulation)	Evening assessment (after stimulation)	Morning assessment (before stimulation)	Evening assessment (after stimulation)
Beta activity	Mean (SD), a.u	0.99 (0.02)	0.76 (0.28)	0.74 (0.27)	0.54 (0.27)	0.57 (0.17)	0.51 (0.07)
	Change from baseline, mean (%)^c^	-	22.8%	25.1%	44.9%	42.0%	48.0%
	Significance^b^	-	0.046	0.12	0.03	0.03	0.03
	Number/percentage of patients with beta activity reduction (%)	-	5/83.3	4/66.6	6/100.0	6/100.0	6/100.0
Theta activity	Mean (SD), a.u.	0.95 (0.10)	0.65 (0.25)	0.60 (0.18)	0.34 (0.32)	0.63 (0.29)	0.43 (0.13)
	Change from baseline, mean (%)^c^	-	29.2%	35.7%	61.2%	32.0%	53.0%
	Significance^b^	-	0.14	0.07	0.07	0.07	0.07
	Number/percentage of patients with theta activity reduction (%)	-	3/75.0	4/100.0	4/100.0	4/100.0	4/100.0
UPDRS total motor score 18-31	Mean (SD)	27.0 (16.2)	20.5 (11.1)	22.0 (10.12)	16.5 (9.4)	17.8 (9.9)	12.0 (8.15)
	Change from baseline, mean (%)^c^	-	18.1	7.2	34.8	24.1	58.0
	Significance^b^	-	0.08	0.11	0.03	0.07	0.03
	Number/percentage of patients showing improvements (%)	-	4/66.6	4/66.6	6/100.0	5/83.3	6/100.0
UPDRS tremor subscore 20-21	Mean (SD)	9.8 (4.9)	6.8 (3.1)	6.8 (2.2)	6.8 (2.9)	6.0 (3.6)	3.5 (1.7)
	Change from baseline, mean (%)^c^	-	23.7	24.3	24.8	38.5	60.4
	Significance^b^	-	0.07	0.04	0.046	0.07	0.03
	Number/percentage of patients showing improvements (%)	-	2/50.0	3/75.0	3/75.0	4/100.0	4/100.0
UPDRS bradykinesia/rigidity subscore 22-26	Mean (SD)	14.8 (9.5)	11.0 (7.0)	12.3 (7.1)	8.2 (5.9)	9.3 (6.6)	6.0 (4.8)
	Change from baseline, mean (%)^c^	-	22.0	13.0	43.5	34.0	63.1
	Significance^b^	-	0.046	0.43	0.01	0.03	0.01
	Number/percentage of patients showing improvements (%)	-	5/83.3	4/63.3	6/100.0	6/100.0	6/100.0

a.u., arbitrary units.

a A positive change from baseline indicates a reduction (i.e., an improvement) in physiological activity and UPDRS motor scores.

b Wilcoxon matched pairs test. Comparison vs baseline.

c Percentual changes were calculated for each patient individually (compared with the individual baseline) and averaged afterward. Positive values indicate a reduction.

The UPDRS motor score averaged over six patients on the morning of the first day was 27.0 ± 16.2 (*P* = 0.72 compared with pre-implantation scores) and served as baseline. In the morning of the third day before stimulation, the UPDRS motor scores were reduced in five patients (mean reduction, 24.1%; *P* = 0.07, n = 6), followed by a more pronounced improvement in all six patients on the third day after stimulation (mean reduction, 58.0%; *P* = 0.03, n = 6; Fig. 1H, M, and Table1). Tremor-related UPDRS motor score subitems 20 to 21 were reduced in all four patients presenting with tremor by an average of 60.4% (*P* = 0.03, n = 4) on the evening of the third day, whereas the UPDRS motor score subitems 22 through 26 (rigidity and bradykinesia) were reduced in all six patients (mean reduction, 63.1%; *P* = 0.01, n = 6), respectively (Table1 and Fig. 1I). Coordinated reset neuromodulation showed significant acute aftereffects (averaged over the 3 stimulation days in all patients, 26.8%, *P* = 0.03). The reduction of UPDRS motor scores positively correlated with the decrease in individual beta band peak power (Fig. 1E; *r* = 0.83; *P* = 0.041; n = 6), whereas theta band power did not show such a correlation. No adverse events were observed.

## Discussion

We show here in PD patients that CR neuromodulation has significant as well as cumulative aftereffects on beta LFP oscillations that were positively correlated with a reduction of motor symptoms. All six patients responded well to CR neuromodulation. Our results are in accordance with theoretical predictions,^5^,^6^ in vitro experiments^9^, preclinical results in MPTP monkeys,^10^ and pathophysiological findings relating abnormal neuronal synchrony to motor signs.^7^,^8^ The CR effects on motor symptoms and abnormal brain oscillations are quite consistent. Beta oscillations^7^,^8^ and corresponding UPDRS subitems 22 through 26 were reduced in all six patients in the morning and evening of the third day. In addition, tremor-related theta LFP oscillations^15^ and UPDRS subitems 20 and 21 were reduced in all four patients with tremor in the morning and evening of the third day.

The electrophysiological and clinical CR effects, observed in all six patients (reduction of LFP beta activity by 48% and of UPDRS motor scores by 58% compared with baseline), were cumulative; that is, after the third daily dose of CR neuromodulation (in the evening of day 3) UPDRS motor scores showed a significant improvement (*P* = 0.03), and beta activity showed a trend toward a significant reduction (*P* = 0.08) compared with the evening of the first day. This cumulative improvement is particularly remarkable, because it is in the opposite direction of the worsening typically observed (personal observations) as a consequence of the fading insertional effect. Despite limitations because of the small sample size, the non-blinded, open-label design, missing high-frequency or placebo comparison, and short treatment duration, our conclusive electrophysiological data, correlated with clinical improvement confirm, previous theoretical^5^,^6^ and experimental^9^,^10^ data and enable one to elaborate effects of CR neuromodulation of the STN in PD patients. The risk of a type I error attributable to multiple statistical comparisons is also present. Of course, the effects of electrical CR neuromodulation have to be confirmed in a randomized controlled trial. However, conducting randomized controlled trials and developing the corresponding implantable device for electrical CR neuromodulation require substantial investments. Proof-of-concept studies such as ours are, hence, key for the overall decision making as well as for the design of future trials. Should appropriate randomized controlled trials ultimately confirm the encouraging preliminary results shown here, electrical CR neuromodulation could represent a substantial advancement for the treatment of Parkinson's disease.
