# Supplementary material for: Coordinated reset neuromodulation for Parkinson's disease: Proof-of-concept study
Source: Mov Disord. 2014 Jun 28;29(13):1679–84. doi: 10.1002/mds.25923 (PMC4282372; doi:10.1002/mds.25923)
Supplement: Supplementary file 1 — Supplementary Information [file mds0029-1679-sd1.docx]

**Supplementary material**

**Study population and surgical procedures.**

Following the prominence of motor symptoms, different clinical subtypes have been defined: akinetic-rigid, tremor-dominant and equivalent. The “akinetic-rigid score” was calculated from the sum of UPDRS items 18 (“speech”), 19 (“facial expression”), 22 (“rigidity”), 23 (“finger taps”), 24 (“hand movements”), 25 (“rapid alternating movements of hands”), 26 (“leg agility”), 27 (“arising from chair”), 28 (“posture”), 29 (“gait”), 30 (“postural stability”) and 31 (“body bradykinesia and hypokinesia”), divided by the number of single sub-items. The “tremor score” was calculated from the sum of UPDRS items 20 (“tremor at rest”) and 21 (“action or postural tremor of hands”), divided by the number of single sub-items. Patients were classified as having **an** akinetic-rigid PD subtype **when the** ‘‘akinetic-rigid score’’ was higher than **the** “tremor score” by **a** factor **of two** or greater. Vice versa, for the tremor-dominant PD subtype, **the** ‘‘tremor score’’ must be at least twice the “akinetic-rigid score.” In our study, no patient was classified as having **the** tremor-dominant PD subtype. **The** equivalent type subgroup included all patients in whom the “tremor score” and “akinetic-rigid” scores differed by less than a factor **of** 2. All patients underwent bilateral implantation of quadripolar macro-electrodes (Medtronic 3389 Medtronic, Inc., Minneapolis, Minnesota, USA) into the STN, performed by senior stereotactic neurosurgeons (MM & VS), as previously described^1^. Stereotactic magnetic resonance images (MRI, T1-T2-weighted) with a resolution of 512x512 pixels and 70 slices (slice thickness: 2 mm) were obtained. The MRI was scaled along the x and y directions to a resolution of 1024x1024 pixels for manual segmentation of the functional target, the STN. The segmentation of the target structure was **performed** by experienced neurosurgeons (MM & VS) with the STP3 stereotactic guidance **software** (Leibinger, Freiburg, Germany). For the exact position of the DBS electrode, all contacts were located and verified separately by postoperative stereotactic orthogonal X-rays. **The analysis of the position of the electrodes with respect to the segmented MRI images revealed that one contact of the macroelectrode was placed within the dorsolateral STN in one patient, two contacts were placed in three patients, and three contacts were placed within the dorsolateral STN in two patients.** To reduce possible insertional effects, that is, local electrophysiological changes and symptom improvement due to local edema and a possible microlesion effect, CR neuromodulation^2^ was started three days after lead implantation and was applied for three consecutive days in two daily sessions for up to two hours (Figure 1A). Because of technical constraints, CR neuromodulation was performed unilaterally, exclusively contralateral to the more severely affected side.

**Coordinated reset neuromodulation**

The repetition rate of CR neuromodulation^2^ (Figure s1) was individually adjusted to dominant peaks in the LFP power spectrum. To this end, the LFP was recorded and analyzed prior to the start of each CR neuromodulation session. Using custom designed software^3^, the frequency of the most prominent peak in the frequency band from 2-35 Hz was selected. If two (or more) peaks of similar amplitude were observed, preference was given to the lower frequency peak because we targeted the low-frequency synchronization, and CR neuromodulation is most effective if delivered at rates corresponding to the frequency of synchronized activity as evaluated from **the** local field potential^2^.

Each CR cycle, that is, each CR stimulation period, included three high-frequency pulse trains composed of 3-5 high-frequency pulses, each separately delivered through one of the three active stimulation contacts, respectively (i.e., three pulse trains per cycle). Three CR cycles (ON-cycles) were followed by two cycles without CR neuromodulation (OFF-cycles). According to computational studies^4^, ON-OFF CR neuromodulation is superior to permanent CR neuromodulation (i.e., without OFF-cycles) **because** desynchronization optimally evolves during the OFF-cycles.

The aim of the brief high-frequency pulse trains was to reset the phases of the targeted neuronal sub-populations and to divide the whole neuronal population into phase-shifted sub-populations, ultimately leading to desynchronization and “unlearning” of pathological synaptic connectivity^2,5,6^. CR neuromodulation was applied through a portable external stimulation and registration device^3^. CR neuromodulation was performed during two stimulation sessions per day with a maximum duration of each stimulation session of two hours. The two sessions of CR neuromodulation were separated by a pause of at least one-hour duration. Daily duration of stimulation varied from 2.2 to 4.0 hours because of practical limitations because patients were lying in bed during the stimulation and registration procedure (patients still had to feel comfortable while being wired during the stimulation procedure). The stimulation amplitudes (intensity: 2.0-4.0 mA) corresponded to the therapeutic window defined as the amplitude interval between the lowest current eliciting clinical effects and the lowest current, which is slightly below the threshold where unwanted side effects are elicited. Intensity in the middle of the therapeutic window was selected to perform CR neuromodulation over three stimulation days. After implantation of the deep brain electrodes and prior to the beginning of the study, a short test stimulation was performed to define the most effective pulse width and the number of the pulses in the pulse train. UPDRS motor scores (sum of subitems 18-31) and LFP recordings were obtained preceding and following CR neuromodulation with a 1-kHz sampling rate at rest (Figure 1A). **LFP recordings were performed at rest in a comfortable supine position and the patients were asked not to perform any movements or speak.** Recordings were visually controlled, **and the** time intervals of the LFP recordings **that were** contaminated by active or passive movement artifacts or epochs during which the patient felt asleep (as detected by visual inspection of the LFP recordings and video recordings of the patients) were excluded from the analysis. The application of CR neuromodulation, UPDRS motor score evaluation (assessed by an experienced study physician) and LFP recordings were performed according to the time schedule illustrated in Figure 1A. The minimal delay between **the** cessation of stimulation and the start of LFP recording was 37 sec, **and it was thus** greater than the 12-sec aftereffect from standard DBS^7^.

**Data analysis**

Off-line analysis was performed with MATLAB (version 7.4, The Mathworks, Natick, MA, USA). LFP activity was represented by the differential activity across two neighboring contacts. Spectra were estimated using discrete Fourier **transformation** by dividing the records into blocks of **the** same duration (8196 points), affording a frequency resolution of approximately 0.12 Hz and averaging across these sections^8^. The analyzed blocks had a 75% overlap. Previous reports indicate that abnormally augmented oscillatory activity in the 8-35 Hz band is associated with bradykinesia and rigidity^7,9-13^ **and that** activity in **the** theta band is associated with Parkinsonian tremor^14^. Therefore, we selected the contact pair that displayed **the** greatest power in the 8-35 Hz band on the first day prior to the first CR neuromodulation session for the analysis of 8-35 Hz activity (denoted as the beta-band) and the contact pair that displayed maximal coherence with the tremor in the tremor frequency band for the analysis of the 3-7 Hz band activity. To this end, coherence between the accelerometer signal and LFPs was calculated for all epochs containing tremor activity and for every patient separately using the standard MATLAB function “mscohere,” subsequently averaging across all epochs of a given patient (1.5-sec sliding window). To find the peak in the beta band, the 8-35 Hz interval was checked using a sliding 1-Hz window, which was moved by steps of 0.5 Hz. The beta peak was confirmed visually and by **controlling** charting and change point analysis, using Change-point Analyzer software (Change-point Analyzer 2.3 shareware program; Taylor Enterprises, Illinois, IA, USA), which was previously used in other studies^10,12,13^. After the beta peak was identified at baseline, the high and low boundaries of the 5-Hz window around this peak were not changed throughout the study; that is, the same contact pair and the same boundaries of the 5-Hz frequency window were analyzed over the three stimulation days. In our patient population (n=6), we found a high beta peak (13-35 Hz) in one patient and a low beta peak (8-13 Hz) in the other five patients. Individual peak beta band power was calculated as the mean power in a 5-Hz interval centered around the selected peak. Frequencies between 8 and 35 Hz that were outside the 5-Hz window were analyzed using 1 Hz windows (e.g., 8 to 9 Hz) to investigate possible changes of the beta power outside the individual peak beta band power or shifts of the synchrony to different frequencies. No significant changes were observed in any of the 1-Hz windows outside the peak beta band power window **throughout** the duration of the study. Thus, **the** individual beta band peak remained stable with respect to frequency throughout the study. Spectral theta power was calculated based on the **entire** band (3-7 Hz).

For each patient, the average peak beta band power and the theta power were calculated before and after stimulation; this was performed for each of the three stimulation days. These values were then normalized in each patient separately by dividing by the maximal value observed **throughout** the three days. Individual normalized spectral power values were then averaged over all **six** patients for statistical analysis. UPDRS and spectral power values obtained in the morning of the first stimulation day served as baseline.

Statistical analysis was performed with STATISTICA 8.0 software (www.statsoft.com). Continuous data are presented by means and standard deviations.

**References**

1. Timmermann L, Braun M, Groiss S, et al. Differential effects of levodopa and subthalamic nucleus deep brain stimulation on bradykinesia in Parkinson's disease. Mov Disord. 2008; 23(2): 218-227.
2. Tass PA. A model of desynchronizing deep brain stimulation with a demand-controlled coordinated reset of neural subpopulations. Biol Cybern. 2003;89(2): 81-88.
3. Hauptmann C, Roulet JC, Niederhauser JJ, et al. External trial deep brain stimulation device for the application of desynchronizing stimulation techniques. Journal of neural engineering 2009;6(6): 066003.
4. Lysyansky B, Popovych OV, Tass PA. Desynchronizing anti-resonance effect of m: n ON-OFF coordinated reset stimulation. Journal of neural engineering 2011;8(3): 036019.
5. Tass PA, Majtanik M. Long-term anti-kindling effects of desynchronizing brain stimulation: a theoretical study. Biol Cybern. 2006;94(1): 58-66.
6. Hauptmann C, Tass PA. Therapeutic rewiring by means of desynchronizing brain stimulation. Biosystems 2007;89(1-3): 173-181.
7. Kuhn AA, Kempf F, Brucke C, et al. High-frequency stimulation of the subthalamic nucleus suppresses oscillatory beta activity in patients with Parkinson's disease in parallel with improvement in motor performance. J Neurosci 2008;28(24):6165-6173.
8. Halliday DM, Rosenberg JR, Amjad AM, [Breeze P](http://www.ncbi.nlm.nih.gov/pubmed?term=Breeze%20P%5BAuthor%5D&cauthor=true&cauthor_uid=8987386), [Conway BA](http://www.ncbi.nlm.nih.gov/pubmed?term=Conway%20BA%5BAuthor%5D&cauthor=true&cauthor_uid=8987386), [Farmer SF](http://www.ncbi.nlm.nih.gov/pubmed?term=Farmer%20SF%5BAuthor%5D&cauthor=true&cauthor_uid=8987386). A framework for the analysis of mixed time series/point process data - Theory and application to the study of physiological tremor, single motor unit discharges and electromyograms. Prog Biophys Mol Biol .1995;64(2-3): 237-278.
9. Eusebio A, Brown P. Synchronisation in the beta frequency-band - The bad boy of parkinsonism or an innocent bystander? Exp Neurol. 2009;217(1): 1-3.
10. Kühn AA, Kupsch A, Schneider GH, Brown P. Reduction in subthalamic 8-35 Hz oscillatory activity correlates with clinical improvement in Parkinson's disease. Eur J Neurosci 2006;23(7):1956-1960.
11. Levy R, Ashby P, Hutchison WD, Lang AE, Lozano AM, Dostrovsky JO. Dependence of subthalamic nucleus oscillations on movement and dopamine in Parkinson's disease. Brain 2002;125:1196-1209.
12. Fogelson N, Pogosyan A, Kuhn AA, et al. Reciprocal interactions between oscillatory activities of different frequencies in the subthalamic region of patients with Parkinson's disease. Eur J Neurosci 2005;22(1):257-266.
13. Kuhn AA, Williams D, Kupsch A, et al. Event-related beta desynchronization in human subthalamic nucleus correlates with motor performance. Brain 2004;127:735-746.
14. Tass P, Smirnov D, Karavaev A, et al. The causal relationship between subcortical local field potential oscillations and Parkinsonian resting tremor. Journal of neural engineering 2010;7(1):016009.

**Figure s1**. **Coordinated reset (CR) neuromodulation.**
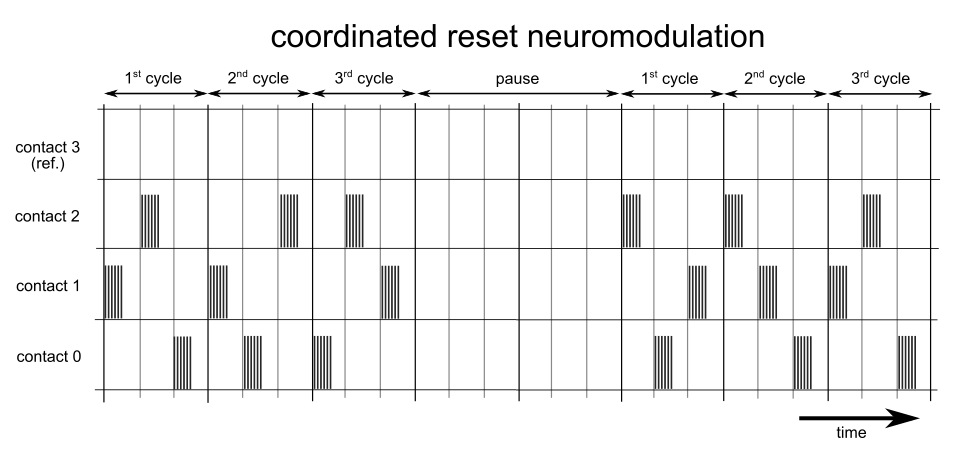


In CR neuromodulation, brief high-frequency pulse trains are delivered through different sites at different, equally spaced times. The 3:2 ON-OFF pattern^2,4^ and the random variation of the pulse train sequences^5^ optimize the desynchronizing CR effect. The pattern is repeated periodically.

**Table s1: Side-specific effects of CR neuromodulation.**

|  | | **Day 1 after stim** | | **Day 2 before stim** | | **Day 2 after stim** | | **Day 3 before stim** | | **Day 3 after stim** | |
| --- | --- | --- | --- | --- | --- | --- | --- | --- | --- | --- | --- |
|  |  | **Absolute reduction from baseline (in points)** | **Relative reduction from baseline (in %)** | **Absolute reduction from baseline (in points)** | **Relative reduction from baseline (in %)** | **Absolute reduction from baseline (in points)** | **Relative reduction from baseline (in %)** | **Absolute reduction from baseline (in points)** | **Relative reduction from baseline (in %)** | **Absolute reduction from baseline (in points)** | **Relative reduction from baseline (in %)** |
| **UPDRS 20-26** | **Change ipsilateral, mean (SD)** | 1.5 (1.7) | 24.3 (29.4) | 0.8 (1.5) | 19.9 (36.8) | 2.1 (2.2) | 40.8 (30.9) | 1.8 (1.8) | 37.1 (35.4) | 3.2 (1.9) | 66.3 (31.1) |
|  | **Change contralateral, mean (SD)** | 2.0 (2.7) | 23.3 (36.6) | 1.7 (1.8) | 19.8 (25.8) | 2.9 (2.6) | 37.0 (22.3) | 2.8 (2.4) | 36.1 (16.1) | 4.8 (2.7) | 64.6 (18.2) |
|  | **Significance ipsilateral vs. contralateral^a^** | 0.63 | 0.75 | 0.42 | 0.81 | 0.62 | 0.75 | 0.57 | 0.87 | 0.57 | 0.20 |
| **UPDRS**  **tremor**  **subscore 20-21** | **Change ipsilateral, mean (SD)** | 1.0 (1.2) | 23.8 (31.6) | 1.0 (0.8) | 40.5 (42.3) | 0.8 (1.7) | 35.7 (47.4) | 1.3 (1.0) | 48.8 (43.7) | 2.0 (1.8) | 64.3 (47.4) |
|  | **Change contralateral, mean (SD)** | 2.0 (2.8) | 27.7 (40.5) | 1.8 (2.1) | 23.7 (28.5) | 2.0 (1.4) | 29.0 (21.3) | 2.3 (1.0) | 34.5 (14.3) | 4.8 (1.9) | 72.1 (26.4) |
|  | **Significance ipsilateral vs. contralateral^a^** | 0.75 | 1.00 | 0.77 | 0.77 | 0.30 | 1.00 | 0.18 | 0.88 | 0.19 | 0.56 |
| **UPDRS**  **bradykinesia/**  **rigidity subscore 22-26** | **Change ipsilateral, mean (SD)** | 1.8 (2.0) | 24.7 (30.9) | 0.7 (2.0) | 6.2 (28.3) | 3.0 (2.1) | 44.2 (18.4) | 2.17 (2.1) | 29.3 (30.5) | 4.0 (1.7) | 67.6 (19.6) |
|  | **Change contralateral, mean (SD)** | 2.0 (3.0) | 20.4 (37.4) | 1.7 (1.9) | 17.2 (26.3) | 3.5 (3.1) | 42.3 (23.3) | 3.17 (3.1) | 37.1 (18.5) | 4.8 (3.3) | 59.6 (10.3) |
|  | **Significance ipsilateral vs. contralateral^a^** | 0.74 | 0.80 | 0.37 | 0.47 | 0.93 | 0.47 | 0.57 | 0.52 | 1.00 | 0.62 |

^a^ Mann–Whitney *U* test.

Although the absolute reduction of the UPDRS subscores (measured in points) was greater on the side contralateral to stimulation, changes from baseline did not differ significantly between contralateral and ipsilateral sides. The lack of significant differences between **the** contralateral and ipsilateral sides may be due to the low number of patients.
